# Barriers to and enablers of uptake of antiretroviral therapy in integrated HIV and tuberculosis treatment programmes in sub-Saharan Africa: a systematic review and meta-analysis

**DOI:** 10.1186/s12981-021-00395-3

**Published:** 2021-11-16

**Authors:** Benjamin Momo Kadia, Christian Akem Dimala, Noah T. Fongwen, Adrian D. Smith

**Affiliations:** 1 Department of Clinical Sciences, Liverpool School of Tropical Medicine, Liverpool, UK; 2 Department of Medicine, Reading Hospital, Tower Health System, West Reading, PA USA; 3 Health and Human Development (2HD) Research Network, Douala, Cameroon; 4 Department of Clinical Research, London School of Hygiene and Tropical Medicine, London, UK; 5 Africa Centres for Disease Control and Prevention (CDC) Innovation Hub, Africa CDC, Addis Ababa, Ethiopia; 6 Nuffield Department of Population Health, University of Oxford, Oxford, UK

**Keywords:** Integrated treatment, Tuberculosis, HIV, Uptake, Barriers, Enablers

## Abstract

**Introduction:**

Programmes that merge management of Human Immunodeficiency Virus (HIV) and tuberculosis (TB) aim to improve HIV/TB co-infected patients’ access to comprehensive treatment. However, several reports from sub-Saharan Africa (SSA) indicate suboptimal uptake of antiretroviral therapy (ART) even after integration of HIV and TB treatment. This study assessed ART uptake, its barriers and enablers in programmes integrating TB and HIV treatment in SSA.

**Method:**

A systematic review was performed. Seven databases were searched for eligible quantitative, qualitative and mixed-methods studies published from March 2004 through July 2019. Random-effects meta-analysis was used to obtain pooled estimates of ART uptake. A thematic approach was used to analyse and synthesise data on barriers and enablers.

**Results:**

Of 5139 references identified, 27 were included in the review: 23/27 estimated ART uptake and 10/27 assessed barriers to and/or enablers of ART uptake. The pooled ART uptake was 53% (95% CI: 42, 63%) and between-study heterogeneity was high (I^2^ = 99.71%, p < 0.001). WHO guideline on collaborative TB/HIV activities and sample size were associated with heterogeneity. There were statistically significant subgroup effects with high heterogeneity after subgroup analyses by region, guideline on collaborative TB/HIV activities, study design, and sample size. The most frequently described socioeconomic and individual level barriers to ART uptake were stigma, low income, and younger age group. The most frequently reported health system-related barriers were limited staff capacity, shortages in medical supplies, lack of infrastructure, and poor adherence to or lack of treatment guidelines. Clinical barriers included intolerance to anti-TB drugs, fear of drug toxicity, and contraindications to antiretrovirals. Health system enablers included good management of the procurement, supply, and dispensation chain; convenience and accessibility of treatment services; and strong staff capacity. Availability of psychosocial support was the most frequently reported enabler of uptake at the community level.

**Conclusions:**

In SSA, programmes integrating treatment of TB and HIV do not, in general, achieve high ART uptake but we observe a net improvement in uptake after WHO issued the 2012 guidelines on collaborative TB/HIV activities. The recurrence of specific modifiable system-level and patient-level factors in the literature reveals key intervention points to improve ART uptake in these programmes.

*Systematic review registration*: CRD42019131933.

**Supplementary Information:**

The online version contains supplementary material available at 10.1186/s12981-021-00395-3.

## Introduction

Among persons living with HIV/AIDS (PLWHA) in low-income settings, tuberculosis (TB) remains the principal cause of mortality [1–3]. In 2017, PLWHA accounted for 900, 000 (9%) of the estimated 10 million new TB disease cases worldwide [4] and of the 900,000 co-infected patients, up to 300,000 (33.3%) died because of TB [5]. The majority of these co-infected patients reside in sub-Saharan Africa (SSA) and according to the World Health Organization (WHO) global TB report of 2018, up to 72% of all patients co-infected with HIV and TB resided in the region [4].

From a therapeutic perspective, low-income settings of SSA initially relied on separate vertical HIV and TB programmes to deliver concurrent HIV and TB treatment to co-infected patients [6–11]. In 2004, based on evidence suggesting that better treatment outcomes are observed when both programmes are integrated, the World Health Organisation (WHO) published policy guidelines to steer the integration of HIV and TB treatment services [12]. Various approaches of delivering integrated services have been proposed and vary from having the services within one health facility to a one-stop-shop strategy in which the services are provided as a single package by the same healthcare team [13]. The 2004 WHO guidelines comprised activities aimed at integrating TB services into HIV care settings with the objective of decreasing the burden of TB in PLWHA and integrating HIV services into TB control programmes with the objective of decreasing the burden of HIV in TB patients [12]. To reduce the burden of TB in PLWHA, WHO recommended intensified TB case-finding, isoniazid preventive therapy and infection control in healthcare settings. To reduce the burden of HIV in TB patients, WHO made an emphasis on HIV counselling and testing and HIV prevention methods for all TB patients, and cotrimoxazole preventive therapy and HIV/AIDS care and support (including ART) for co-infected patients [12]. It is worth noting that these initial guidelines were based on incomplete evidence and were therefore meant to serve as provisional guidelines [14].

In 2012, WHO issued a review of the 2004 guidelines [14]. Overall, the updated policy employs the same framework as the interim policy but emphasized on strategies for delivering integrated HIV/TB treatment, preferably at the same time and location. The new guidelines encourage the establishment of mechanisms of delivering both HIV and TB treatment within other programmes such as maternal and child health, and prison health services [14]. Furthermore, monitoring and evaluation of activities linked with integrated HIV/TB treatment are expected to be based on standardized indicators and reporting formats. In this light, it is worth noting that uptake of and adherence to treatment are important indicators of the quality and therapeutic outcomes of integrated care [14]. WHO recommends that HIV-infected TB patients should be initiated on ART irrespective of their CD4 count, as timely initiation of ART during TB therapy has been shown to significantly improve survival [15]. ART should be started within 8 weeks of initiation of anti-TB treatment and in TB patients with a CD4 count of less than 50cells/mm3, ART should be started within 2 weeks after the onset of anti-TB treatment [15–17]. ART is associated with severe adverse events in HIV patients with TB meningitis so ART in these cases should be delayed. In the event of TB-associated immune reconstitution inflammatory syndrome (IRIS), anti-TB and ART should be continued as IRIS is typically self-limiting [18–20].

Reports on integrated TB/HIV treatment services in SSA have tended to focus on quantitative data on coverage and functionality, with scarce exploration of factors affecting ART uptake which is an important indicator of the success of integrated TB/HIV treatment. The need to investigate this indicator in SSA is further supported by compelling reports from the region which suggest suboptimal uptake of antiretroviral therapy (ART) even when HIV and TB treatment services are integrated [21, 22]. This study sought to estimate ART uptake in programmes integrating treatment of HIV and TB in SSA and to summarize existing evidence on the barriers to and facilitating factors for the uptake of ART in these programmes.

## Methods

### Search strategy

Medline, Embase, Cochrane, Popline, Scopus, Global Health and Africa journal online databases were searched extensively to include peer-reviewed studies published from March 2004 (when WHO first issued recommendations governing integrated HIV/TB care) through July 2019. The search terms and their variations that were used in combination are shown on Additional file 1. Articles retrieved from the search were exported to and saved on Mendeley desktop software version 1.19.8.0.

### Selection of studies

Two investigators (BMK and CAD) independently screened the titles and/or abstracts and when necessary, the full texts of studies identified using the search strategy, to assess each study’s eligibility for inclusion. The assessments were made using selection criteria that were developed from clearly defined study population (participants), intervention and outcomes of interest, study design, research methods, setting and language. The inclusion criteria were as follows: randomized trials and observational (cross-sectional and cohort) studies published in English language; studies using quantitative, qualitative, or mixed research methods; studies describing integrated treatment of HIV and TB (the delivery of both HIV and TB treatment at the same time, by the same health team, at the same location) in sub-Saharan Africa; studies involving adults newly diagnosed with HIV/ pulmonary TB co-infection or pulmonary TB patients on anti-TB drugs who are newly diagnosed with HIV and require ART; studies involving healthcare providers and other stakeholders involved in the delivery of integrated HIV/TB treatment; studies with at least one of the outcomes of interest (ART uptake, barriers to ART uptake, or enablers of ART uptake). Exclusion criteria were as follows: studies involving TB patients only or HIV infected persons only; studies involving other forms of TB other than pulmonary TB; studies involving delivery of only ART or only anti-TB drugs; and studies in which HIV and TB treatment are delivered via separate vertical programmes. Conference abstracts, editorials, policy briefs, policy discussions, bulletins, grey literature, and letters to editors were excluded. The reference lists of eligible articles were hand searched to identify other eligible studies.

### Assessment of methodological quality

BMK and CAD independently assessed the methodological quality of the studies. The quality of qualitative studies was graded using the Critical Appraisal Skills Programme [23] while those of interventional and observational studies were assessed using their respective quality assessment tools as per the National Health Institute (National Heart, Lung, and Blood Institute) [24]. For mixed-methods studies, the qualities of the qualitative and quantitative components were separately assessed using appropriate tools as described above.

### Data extraction

Data extraction forms and definitions of key terms were developed to standardise the data collection process. Two extraction forms were developed on Microsoft Excel 2016: one for qualitative data (barriers and enablers) and one for quantitative data (uptake of ART). Each of the forms was piloted using three studies. BMK and CAD independently extracted relevant data from each retained article and saved the data on the Microsoft Excel forms. Data entered in the forms was then harmonised and subsequently double-checked for accuracy by a third investigator (NFT). Quantitative data was exported to STATA 15. Data on publication details and outcomes of interest were extracted. Data on publication details included first author name, publication year, journal reference, country and place of study, year of study, study design, study area and setting, study population, sample size, characteristics of patients (such as age and sex distribution etc.), ART initiation policy and WHO TB/HIV management policy, as well as limitations and strengths of studies. ‘ART initiation policy’ was considered a binary variable with the categories being initiation prior to the test and treat policy of 2015 (ART initiation depended on CD4 count) versus initiation following the test and treat policy guidelines of 2015. ‘WHO TB/HIV management policy’ was also considered a binary variable with the categories being treatment following 2004 guidelines versus treatment following 2012 guidelines. Data on outcomes of interest included ART uptake (measured as the reported proportion of persons diagnosed HIV seropositive and found eligible for HIV treatment and who effectively initiated ART; from 2015 when universal ART was recommended, all persons diagnosed HIV seropositive were eligible to initiate ART), barriers to ART uptake and enablers of ART uptake. For studies providing estimates on ART initiation, when a study provided data spanning different policy periods or timings of ART initiation, we recorded and analysed the data for each period or timing separately. For qualitative studies, specific barriers and enablers were extracted as reported in the studies. As concerns quantitative studies investigating factors associated with uptake of ART, factors associated with poor uptake were considered as barriers while factors associated with good uptake were considered as enablers.

### Data analysis and synthesis

A thematic approach was used to analyse and synthesise data on barriers to and enablers of uptake of ART. This approach was also employed for studies that quantitatively assessed factors associated with ART uptake. BMK and CAD developed the initial coding framework on Microsoft Excel 2016 by reading through the results of all the eligible studies to identify the main themes. Based on the initial coding, broad themes were developed under which all text units were iteratively grouped into one of the broad themes. Each theme and the text units were then further analyzed and amended to develop more themes and sub-themes.

Meta-analysis was used to obtain the pooled estimate for ART uptake. ART uptake was represented on a forest plot and the risk of publication bias was assessed using a funnel plot. The degree of between-study variability in the ART uptake was assessed by visual inspection of the forest plots and interpretation of the *I*^2^ statistic from meta-analysis (none (I^2^ < 25%), low (25 ≤ I^2^ ≤ 49%), moderate (50 ≤ I^2^ ≤ 74%) or high (I^2^ ≥ 75%). The p-value for heterogeneity was used to determine whether the between-study variability is associated with variations in the size of ART uptake across studies. Pooled estimates of ART uptake categorized by variables such as study design, study population size, region, study setting, and WHO guideline on collaborative TB/HIV activities were also assessed. Meta-regression was used to investigate possible associations between these variables and between-study heterogeneity in ART uptake.

This study has been reported as per the Preferred Reporting Items for Systematic Reviews and Meta-Analysis (PRISMA) guidelines (Additional file 2). The protocol for this review was registered with the Prospective Registry of Systematic Reviews (PROSPERO) and the registration number is CRD42019131933.

## Results

### Overview of search output

**Fig. 1:**
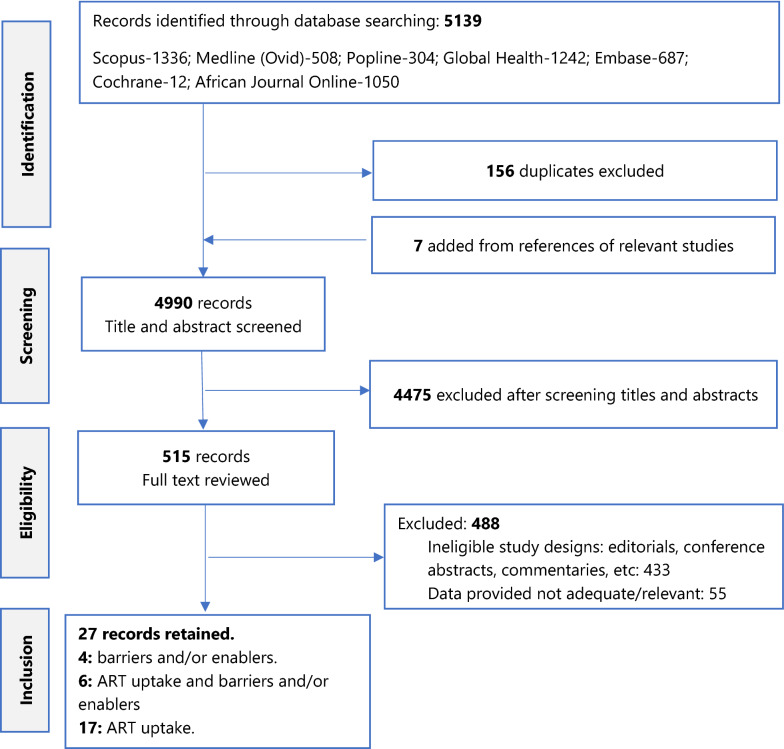
PRISMA flowchart showing process of selection of studies that were reviewed in the systematic review of barriers to and enablers of uptake of antiretroviral therapy in the context of integrated HIV and tuberculosis treatment among adults in sub-Saharan Africa

The search yielded a total of 5139 articles. Figure 1 is a PRISMA flowchart detailing the process by which we arrived at the final list of 27 studies. After removing duplicates, screening the titles and abstracts of the articles, excluding ineligible articles, and manually searching the reference lists of eligible articles, a harmonised list of 27 studies with one of more outcomes of interest was retained for analysis: 4 assessed barriers and/or enablers of ART uptake, 17 estimated ART uptake and 6 assessed both ART uptake and barriers and/or enablers. Overall, twenty-three (23/27) studies estimated ART uptake and involved 22847 TB/HIV co-infected patients, but the analysis was conducted on 21630/22847 (94.7%) participants who had full relevant data. Thirteen (14/23) of the studies were from East Africa, 4/23 from Southern Africa and 3/23 from Central Africa and 2/23 from West Africa. Table 1 summarizes these studies.

**Table 1:** Studies with estimates of ART uptake in the context of integrated HIV/TB treatment among adults in SSA

	Author, year	Study setting	Study design	HIV/TB co-infected	Participants with required data	Uptake (%)
1	Burnett, 2018	Rural Uganda	Cross-sectional	179	103	47.7
2	Ferroussier, 2013	Rural and urban Benin	Retrospective cohort	1255	1111	42
3	Gasana, 2008	Rural Rwanda	Prospective cohort	125	125	43.2
4	Harris, 2008	Urban Zambia	Prospective cohort	1006	1006	59
5	Shaffer, 2011	Rural Kenya	Secondary analyses of retrospective cohort data	1911	1911	35.8
6	Simieneh, 2017	Urban Ethiopia	Cross-sectional	246	246	31.7
7	Takarinda, 2012	Rural and urban Zimbabwe	Retrospective cohort study	2655	2655	42
8	Tweya, 2014 (before implementation of 2011 Malawi national ART/PMTCT guidelines)	Urban Malawi	Retrospective cohort	377	377	70
9	Tweya 2014 (after implementation of 2011 Malawi national ART/PMTCT guidelines)	Urban Malawi	Retrospective cohort	308	308	78
10	Van Rie, 2014	Urban DRC	Prospective cohort study	513	513	69
11	Kumwenda, 2011	Rural and urban Malawi	Retrospective cohort	996	996	38.1
12	Pepper, 2011	Urban slum, South Africa	Secondary analysis of prospective cohort data	100	100	66
13	Ndagijimana, 2015 (Setting of Kicukiro)	Urban Rwanda	Retrospective cohort	352	352	71
14	Ndagijimana, 2015 (Setting of Rulindo)	Rural Rwanda	Retrospective cohort	60	60	93.8
15	Njozing, 2010	Urban and rural Cameroon	Retrospective cohort	1473	1220	50.3
16	Patel, 2014	Urban DRC	Prospective cohort	599	492	82.5
17	Hermans, 2012	Urban Uganda	Retrospective cohort	366	228	57
18	Huerga, 2010 (short-term after integration)	Rural Kenya	Retrospective cohort	437	325	46.2
19	Huerga, 2010 (medium term after integration)	Rural Kenya	Retrospective cohort	477	332	41.3
20	Kanyerere, 2012	Urban Malawi	Retrospective cohort	1190	1190	57.8
21	Ledibane, 2015	Rural and urban South Africa	Cross-sectional	2761	2761	35.9
22	Owiti, 2015	Rural Kenya	Retrospective cohort	323	323	60.7
23	Pathmanathan, 2018	Rural and urban Swaziland	Retrospective cohort	466	277	98.9
24	Ansa, 2014	Urban Ghana	Prospective and retrospective cohort	132	79	59.5
25	Van Lettow, 2011 (before policy to start ART as early as 2 weeks after commencing treatment for active TB	Urban Malawi	Prospective cohort study	1111	1111	21.2
26	Van Lettow, 2011 (after policy to start ART as early as 2 weeks after commencing treatment for active TB)	Urban Malawi	Prospective cohort study	1072	1072	28.5
27	Phiri, 2011 (ART started during first 2 months of TB treatment: 2008 reporting period)	Urban Malawi	Case study approach	1219	1219	17
28	Phiri, 2011 (ART started during first 2 months of TB treatment: 2009 reporting period)	Urban Malawi	Case study approach	1138	1138	38
				22,847	21,630	

*ART* antiretroviral therapy, *PMTCT* prevention of maternal-to-child transmission, *SSA* sub-Saharan Africa

Overall, ten (10/27) studies provided data on barriers to and/or enablers of uptake of ART. The data was obtained from a total of 3514 participants and 12 focus group discussions. Six (6/10) of the studies were conducted in East Africa, 2/10 in South Africa, 1/10 in West Africa and 1/10 in Central Africa. Table 2 summarizes the studies.

**Table 2:** Studies with data on barriers to and/or enablers of ART uptake in the context of integrated HIV/TB treatment in sub-Saharan Africa

Author, year	Setting	Research method/ Study design	Participants	Sample size
Kumwenda, 2011	Rural and urban Malawi	Mixed methods: historical cohort study (quantitative) and cross-sectional survey with IDIs (qualitative)	996 HIV/TB co-infected patients 99 newly registered TB patients for IDIs	996-quantitative 99-qualitative
Patel, 2014	Urban DRC	Quantitative: Prospective cohort	HIV/TB co-infected patients	492
Pepper, 2011	Urban slum, South Africa	Quantitative: Secondary analysis of prospective cohort data	HIV/TB co-infected patients	100
Levin, 2006	Urban South Africa	Mixed-methods cross-sectional study using semi-structured questionnaire with qualitative fields	HIV/TB co-infected persons of predominantly low socioeconomic status	85
Nansera, 2010	Rural Uganda	Mixed methods: cross-sectional study including key informant interviews for qualitative data	Workers of 22 health units	88
Njozing, 2010	Rural and urban Cameroon	Quantitative: retrospective cohort	Staff of hospitals providing TB/HIV treatment and support services	1220
Ndagijimana, 2015	Rural and urban Rwanda	Mixed methods: Quantitative component: Retrospective cohort Qualitative component: cross-sectional with data collection via IDIs and FGDs	-Staff of health facilities (IDIs) -HIV/TB co-infected patients (FGDs)	24 IDIs and 12 FGDs
Wajanga, 2014	Urban Tanzania	Qualitative: Cross-sectional with data collection via IDIs	Hospital staff including administrators, laboratory technicians, pharmacists, and physicians	26 IDIs
Chileshe, 2010	Rural Zambia	Qualitative: ethnographic case-studies	HIV/TB co-infected patients and their households	7 case studies
Tweya, 2014	Urban Malawi	Quantitative: retrospective cohort	HIV/TB co-infected patients	377

*IDI* In-depth interview, *FGD* focus-group discussion, *DRC* democratic Republic of Congo

### Methodological quality of studies

Nineteen of the 23 (19/23) studies with estimates of ART uptake were of moderate quality (Table 3). Table 3 also includes the quality assessment of three studies [22, 31, 34] that used quantitative methods to assess factors associated with (barriers to and/or enablers of) ART uptake.

**Table 3:** Quality appraisal of studies with quantitative estimates of outcomes

Study	Clear research question(s)/ objectives	Clearly defined study population	Participation rate ≥ 50%	Selection from same population	Justified sample size and study power	Exposures measured prior to outcomes	Reasonable timeframe for effect	Different levels of exposure	Clearly defined exposure measures	Repeated measure of exposure	Clearly defined outcomes	Blinded outcome assessors	Loss to follow-up ≤ 20%	Measured and adjusted confounders	Overall rating
Burnett, 2018	Yes	Yes	No	No	Yes	Yes	Yes	Yes	Yes	No	Yes	Can’t tell	No	Yes	Moderate
Ferroussier, 2013	Yes	Yes	Yes	No	Yes	Yes	Yes	No	Yes	No	Yes	Can’t tell	Yes	No	Moderate
Gasana, 2008	No	Yes	Yes	Yes	Yes	Yes	Yes	No	Yes	No	No	Can’t tell	Yes	No	Moderate
Harris, 2008	No	Yes	Yes	No	Yes	Yes	Yes	No	Yes	No	No	Can’t tell	No	No	Moderate
Shaffer, 2011	Yes	Yes	Yes	Yes	Yes	Yes	Yes	No	Yes	No	Yes	Can’t tell	Yes	No	Moderate
Simieneh, 2017	Yes	Yes	Yes	Yes	Yes	Yes	Yes	No	Yes	No	Yes	Can’t tell	Yes	No	Moderate
Takarinda, 2012	Yes	Yes	Yes	Yes	Yes	Yes	Yes	No	Yes	No	Yes	Can’t tell	Yes	No	Moderate
Tweya, 2014	Yes	Yes	Yes	Yes	Yes	Yes	Yes	Yes	Yes	No	Yes	Can’t tell	Yes	No	Good
Van Rie, 2014	Yes	Yes	Yes	No	Yes	Yes	Yes	No	Yes	No	Yes	Can’t tell	Yes	No	Moderate
Kumwenda, 2011	Yes	Yes	Yes	Yes	Yes	Yes	Yes	No	Yes	No	Yes	Can’t tell	Yes	No	Moderate
Pepper, 2011	Yes	Yes	Yes	Yes	Yes	Yes	Yes	No	Yes	No	Yes	Can’t tell	Yes	Yes	Good
Ndagijimana, 2015	Yes	Yes	Yes	No	Yes	Yes	Yes	No	Yes	No	Yes	Can’t tell	Yes	No	Moderate
Njozing, 2010	Yes	Yes	Yes	No	Yes	Yes	Yes	No	Yes	No	Yes	Can’t tell	Yes	No	Good
Patel, 2014	Yes	Yes	Yes	No	Yes	Yes	Yes	No	Yes	No	Yes	Can’t tell	Yes	Yes	Moderate
Hermans, 2012	Yes	Yes	Yes	Yes	Yes	Yes	Yes	No	Yes	No	Yes	Can’t tell	Yes	No	Moderate
Huerga, 2010	Yes	Yes	Yes	Yes	Yes	Yes	Yes	Yes	Yes	No	Yes	Can’t tell	Yes	No	Moderate
Kanyerere, 2012	Yes	Yes	Yes	Yes	Yes	Yes	Yes	No	Yes	No	Yes	Can’t tell	Yes	No	Moderate
Ledibane, 2015	Yes	Yes	Yes	No	Yes	Yes	Yes	No	Yes	No	Yes	Can’t tell	Yes	No	Moderate
Owiti, 2015	Yes	Yes	Yes	No	Yes	Yes	Yes	Yes	Yes	No	Yes	Can’t tell	Yes	No	Moderate
Pathmanathan, 2018	Yes	Yes	Yes	No	Yes	Yes	Yes	Yes	Yes	No	Yes	Can’t tell	Yes	No	Moderate
Ansa, 2014	Yes	Yes	Yes	Yes	Yes	Yes	Yes	No	Yes	No	Yes	Can’t tell	Yes	No	Good
Van Lettow, 2011	Yes	Yes	Yes	No	Yes	Yes	Yes	Yes	Yes	No	Yes	Can’t tell	Yes	No	Moderate
Phiri, 2011	Yes	Yes	Yes	Yes	Yes	Yes	Yes	Yes	Yes	No	Yes	Can’t tell	Yes	No	Moderate

N/B: The table also includes the three studies [22, 31, 34] that quantitatively assessed factors associated with (i.e. barriers and/or enablers of) ART uptake

The other seven of the ten (7/10) studies that assessed barriers to and/or enablers of ART uptake used qualitative methods to assess the study outcomes and three-quarters of these studies were of moderate quality (Table 4).

**Table 4:** Quality appraisal of qualitative studies assessing barriers and/or enablers

Criteria	Kumwenda, 2011	Levin, 2006	Nansera, 2010	Njozing, 2011	Ndagijimana, 2015	Wajanga, 2014	Chileshe, 2010
1. Was there a clear statement of the aims of the research?	Yes	Yes	Yes	Yes	Yes	Yes	Yes
2. Is a qualitative methodology appropriate?	Yes	Yes	Yes	Yes	Yes	Yes	Yes
3. Was the research design appropriate to address the aims of the research?	Yes	Yes	Yes	Yes	Yes	Yes	Yes
4. Was the recruitment strategy appropriate to the aims of the research?	Yes	Yes	Yes	Yes	Yes	Yes	Yes
5. Was the data collected in a way that addressed the research issue?	Yes	Yes	Yes	Yes	Yes	Yes	Yes
6. Has the relationship between researcher and participants been adequately considered?	No	No	No	No	No	No	No
7. Have ethical issues been taken into consideration?	Yes	Yes	Yes	Yes	Yes	Yes	Yes
8. Was the data analysis sufficiently rigorous?	Yes	Can’t tell	Can’t tell	Yes	Yes	Yes	Yes
9. Is there a clear statement of findings?	Yes	Yes	Yes	Yes	Yes	Yes	Yes
10. How valuable is the research?	Very	Very	Very	Very	Very	Very	Very
Overall risk of bias	Minimal	Moderate	Moderate	Moderate	Moderate	Moderate	Moderate
Overall Rating/Comment	Good	Fair	Fair	Good	Good	Good	Good

### ART uptake

**Fig. 2:**
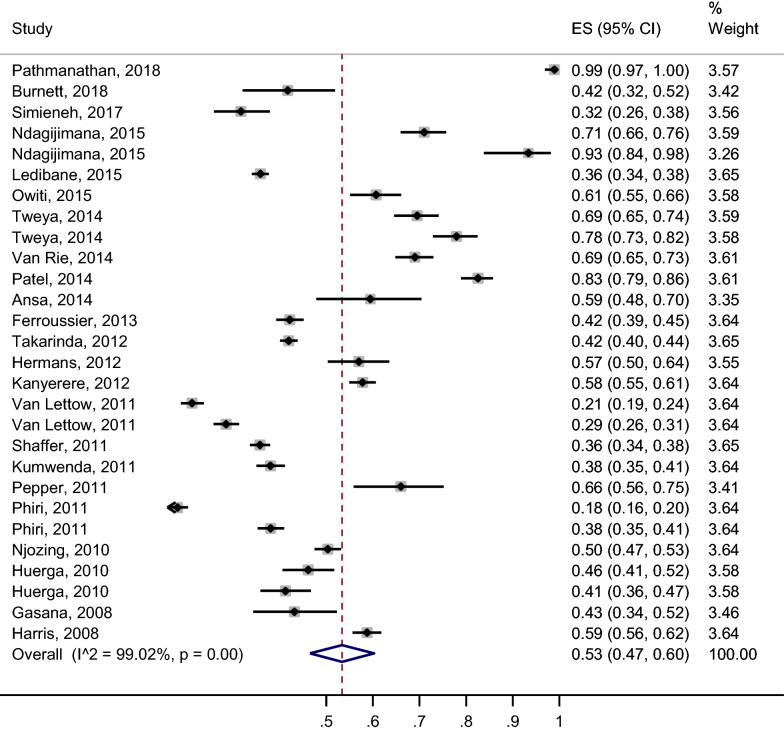
Forest plot showing individual study and pooled estimates of ART uptake. The dashed line on the Forest plot represents the overall pooled estimate. The grey squares and horizontal lines represent the individual study ART uptakes and their 95% confidence intervals. The size of the grey square represents the weight contributed by each study in the meta-analysis. The diamond represents the pooled estimate and its 95% confidence intervals

A total of 28 estimates were obtained from the 23 studies that estimated ART uptake. The individual study estimates of uptake and the reasons for having 28 estimates from 23 studies are detailed on Table 1. Some reasons for the discrepancy included: same study evaluating uptake at two different periods of implementing the integration policy [25], study reporting of uptake at different facilities [26], and study evaluating ART initiation in cohorts with different timings of ART initiation [27]. Uptake of ART ranged from 18.0 to 98.9%, with a pooled estimate of 53% (95% CI: 42, 63%; I^2^ = 99.71%, p < 0.001) and high between-study heterogeneity (Fig. 2). The proportion of between-study heterogeneity explained by sample size (< 1000 versus 1000 or more) was 28.26%; p-value = 0.002 (p < 0.05), indicating strong evidence of an association between sample size and between-study variability in ART uptake. The proportion of between-study heterogeneity explained by WHO guideline on collaborative TB/HIV activities (interim versus revised guidelines) was 14.09%; p-value = 0.031 (p < 0.05), indicating much weaker evidence of an association between the covariate ‘WHO guideline on collaborative TB/HIV activities’ and between-study variability in ART uptake. The proportion of between-study heterogeneity explained by setting of study (categorized as rural, urban, rural and urban) was − 4.82%; p-value = 0.840 (> 0.05), indicating no evidence of an association between study setting and between-study variability in ART uptake.

The results of subgroup analyses are shown on Additional files 3, 4, 5, 6, 7. There was no statistically significant difference in pooled ART uptake stratified by study setting i.e., urban versus rural versus urban and rural (Additional file 3). As shown in Additional file 4, subgroup analysis by region revealed statistically significant subgroup effects (p = 0.021). Pooled ART uptake in Southern Africa and Central Africa were comparable (68%, 95% CI: 47, 86 and 65%, 95% CI: 42, 84% respectively) and much higher than the pooled estimates from East Africa (49%, 95% CI: 41, 57%) and West Africa (43%, 95% CI: 40, 46%). Pooled ART uptake for studies under the revised WHO guidelines on collaborative TB/HIV activities (67%, 95% CI: 52, 80%) was much higher than that for studies based on the 2004 interim guidelines (47%, 95% CI: 40, 53%) as shown in Additional file 5. The subgroup effect was statistically significant (p = 0.013). Additional file 6 shows very strong evidence of a statistically significant subgroup effect with subgroup analysis by study design (p < 0.0001). The highest pooled uptake was in retrospective studies (60%, 95% CI: 52, 68%). The pooled estimate for prospective cohort studies was similar to the overall pooled ART uptake (53%, 95% CI: 34, 72%). As shown in additional file 7, the pooled ART uptake for studies with sample size < 1000 (63%, 95% CI: 52, 74%) was much higher than that of studies with sample ≥1000 (39%, 95% CI: 32, 46%). There was very strong evidence of a statistically significant subgroup effect (p < 0.0001). There was high heterogeneity in all the subgroup analyses.

**Fig. 3:**
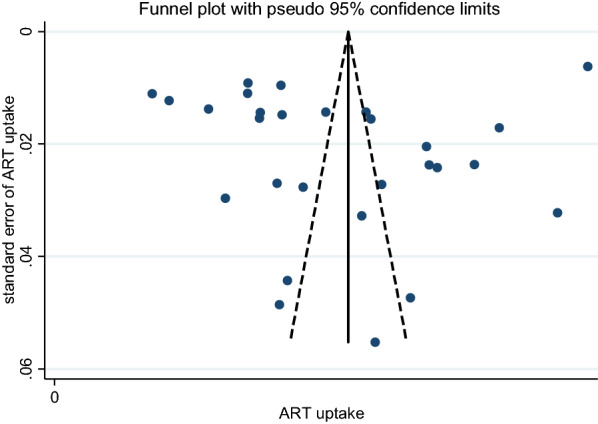
Funnel plot to assess publication bias across studies reporting ART uptake among adults enrolled for integrated treatment of TB and HIV in SSA.

Figure 3 shows an assessment of the risk of publication bias across the estimates of ART uptake. It reveals a low risk of publication bias.

### Barriers to and enablers of ART uptake

The barriers to and enablers of ART uptake are presented in Table 5.

**Table 5:** Barriers and enablers of uptake of antiretroviral therapy in the context of integrated therapy for TB and HIV in SSA

Barriers to ART uptake	
Health system-related	
Limited staff capacity	
Shortage of staff	Nansera, 2010, Chileshe, 2010; Ndagijimana, 2015
Staff turnover after training	Ndagijimana, 2015
Insufficient knowledge and skills on integrated treatment	Nansera, 2010
Limited medical supplies e.g. drug stock-outs/insufficiency	Kumwenda, 2011; Nansera, 2010; Wajanga, 2014
Lack of infrastructure for provision of integrated services	Nansera, 2010; Ndagijimana, 2015
Long enrolment process	Chileshe, 2010; Wajanga, 2014
Poor adherence to or lack of treatment guidelines	Nansera, 2010; Wajanga, 2014
Prerequisite of a guardian during initiation	Kumwenda, 2011
Insufficient staff motivation	Ndagijimana, 2015
Limited HIV status disclosure patterns	Chileshe, 2010
Provider failing to offer ART to patient on anti-TB	Kumwenda, 2011
Delayed initiation because of high CD4	Kumwenda, 2011
Poor integration of inpatient and outpatient HIV and TB Wajanga, 2014 care which limits availability of essential services for inpatients	
Clinical	
Fear of drug toxicity	Kumwenda, 2011
Contraindication to ≥ 1 antiretroviral drug(s)	Kumwenda, 2011
Intolerance to anti-TB drugs	Patel, 2014

Socioeconomic and individual level	
Stigma	Wajanga, 2014; Chileshe, 2010; Levin, 2006
Low socioeconomic status leading to financial constraints such as lack of money for transport to treatment facility	Kumwenda, 2011; Chileshe, 2010
Younger age group	Kumwenda, 2011; Pepper, 2011
Male gender	Pepper, 2011
Denial of HIV status	Chileshe, 2010,
Failure to disclose status to provider	Patel, 2014
Poor social support network	Pepper, 2011
Negative coping such as use of alternative therapies e.g. witchcraft and faith healing	Chileshe, 2010
Fear of HIV testing	Chileshe, 2010
Pill burden	Kumwenda, 2011
Sero-discordant HIV-negative partner	Chileshe, 2010

Enablers of ART uptake	
Health system-related	
Strong staff capacity	
Enough staff for service delivery	Nansera, 2010
Continuing education of staff	Wajanga, 2014,
Equipping staff with adequate knowledge, skills, and mentorship	Nansera, 2010,
Promotion of positive multidisciplinary team approach in treatment	Wajanga, 2014, Njozing, 2011
Re-enforcement of procurement, supply, and dispensation	
Sufficient medical supplies	Nansera, 2010; Wajanga, 2014
Ease policy to allow concurrent counseling and drug administration	Wajanga, 2014
Providers strongly recommend and effectively prescribe drugs	Kumwenda, 2011
Partnership with treatment partners and peer counsellors	Wajanga, 2014
Designate teams for drug administration on weekends/urgent circumstances	Wajanga, 2014
ART for TB/HIV co-infection delivered within ART/PMTCT service	Tweya, 2014
Schedule ART at 2 months less likely to experience delay	Patel, 2014
Convenience and accessibility of services	Ndagijimana, 2015; Njozing, 2011; Levin, 2006
Efficiency and quality in service delivery	Ndagijimana, 2015
Enrolment higher in public compared to faith-based hospitals	Njozing, 2011
Community level	
Availability of psychosocial support Persons living with HIV group facilitating linkage to treatment, performing home visits, providing counseling, and fighting stigma	Chileshe, 2010; Njozing, 2011
Support and motivation from family or friends	Chileshe, 2010
Clinical	
Being a retreatment patient	Tweya, 2014
Patient in HIV care at the start of TB treatment	Tweya, 2014

#### Barriers to uptake of ART

The themes that emerged from the studies with data on barriers were socioeconomic and individual level, health system, and clinical. The most frequent barriers under the socioeconomic and individual level theme were stigma (3 studies) [28–30];poverty/low income (2 studies) [21, 31] and younger age group (2 studies) [21, 31]. Concerning stigma, Levin et al.reported that all patients who declined from attending integrated TB/HIV services mentioned negative stigma surrounding HIV as their principal concern (perceived stigma) [29]. Wajanga et al. also reported perceived stigma as a barrier to HIV status disclosure and ART uptake in TB patients [30].Chileshe et al.described both perceived and enacted stigma which made it difficult for TB patients infected with HIV to disclose their HIV status and access ART [28]. The patients feared gossip and verbal insults (perceived stigma). They were isolated within households, neglected, visited less by relatives and friends, and experienced insults or gossip (enacted stigma). Regarding poverty/low income, it led to lack of transport fare to travel to treatment centres especially when patients had to be accompanied as reported by Kumwenda et al. and Chileshe et al.[21, 28]. As concerns younger age, Kumwenda et al.found that patients aged 15–24 years were least likely to initiate ART [21] while Pepper et al.reported that age less than 36 years was associated with not initiating ART [31]. Other socioeconomic and individual level barriers such as non-disclosure of HIV status to healthcare providers [22] and lack of social support network[31] are outlined in Table 5.

The most common subthemes under the health system related barriers were limited staff capacity (3 studies) [26, 28, 32]; shortages in medical supplies (3 studies) [21, 26, 30]; lack of infrastructure for the provision of integrated treatment services (2 studies) [26, 32]; poor adherence to or lack of treatment guidelines (2 studies) [30, 32]; and long enrolment process [28, 30]. Limited staff capacity presented as shortage of staff (Nansera et al. [32], Chileshe et al. [28], and Ndagijimana et al. [26]), insufficient skills and knowledge on integrated treatment among staff (Nansera et al.) [32] and turnover of staff trained on integrated treatment (Ndagijimana et al.) [26]. Limited medical supply resulted from drug stock-outs or insufficiency as revealed by Kumwenda et al. [21]*,* Wajanga et al. [30] and Nansera et al. [32]. Other health system related barriers such as poor staff motivation (Ndagijimana et al.) [26] and the provider failing to offer ART to TB patients (Kumwenda et al.) [21]. Clinical challenges were essentially drug-related and included intolerance to anti-TB drugs (Patel et al.) [22], providers’ fear of drug toxicity (Kumwenda et al.) [21]; and contraindications to one or more antiretrovirals (Kumwenda et al.) [21].

#### Enablers of uptake of ART

Health system and community level factors were the most frequently reported enablers of uptake. The most frequently described health system related enabling factors were re-enforcement of procurement, supply, and dispensation chain (5 studies) [21, 22, 26, 30, 32]; convenience and accessibility of treatment services (3 studies) [26, 29, 34]; and strong staff capacity (3 studies) [30, 32, 34]. Some strategies to strengthen procurement, supply and dispensation included ensuring availability of sufficient medical supplies [30, 32], effective prescription of drugs by providers[21], and designating teams to provide drugs at odd times such as weekends [30]. Wajanga et al. reported that easing policy to allow concurrent counselling and drug administration instead of separating the two procedures could facilitate ART initiation [30]. In the qualitative study by Ndagijimana et al., providers reported that delivering treatment of TB and HIV at once and at the same location enhanced quality of service and this was acknowledged by patients [26]. Other strategies are mentioned in Table 5. As concerns strengthening of staff capacity, Nansera et al. reported equipping staff with adequate knowledge/skills and ensuring there are enough staff for service delivery [32] while Wajanga et al. reported continuing education of staff and promoting positive multidisciplinary approach to patient management [30]. Njozing et al. also reported use of multidisciplinary team approach [35]. As for convenience and accessibility of services, studies by Njozing et al. [35]*,* Ndagijimana et al. [26]*,* and Levin et al. [29] revealed that joint treatment services for TB and HIV was more convenient to patients and eased patients’ access to treatment which facilitates treatment uptake.

Availability of psychosocial support (2 studies) [28, 35] was the most frequently reported enabler of ART uptake at the community-level. The reported types of psychosocial support were in the form of persons living with HIV support group (Chileshe et al. [28], Njozing et al. [35]) and informally through support from family or friends (Chileshe et al.) [28]. Chileshe et al. reported how an HIV support group helped link a patient to treatment [28]. Njozing et al. reported a variety of roles of HIV support groups including counselling and home visits to the sick [35]. Clinical factors enabling uptake were described in one study and included being a retreatment patient and being in HIV care at the start of TB treatment (Tweya et al.) [33]

## Discussion

Co-delivery of TB and HIV treatment services within the same location is expected to enhance the control of TB and HIV co-pandemics by facilitating patients’ access to treatment commodities and ensuring efficiency in treatment delivery [36]. It has even been reported that patients offered full integration of TB and HIV treatment services are more likely to initiate ART than those not receiving integrated treatment [37]. Despite the known merits of integrating treatment of TB and HIV, coupled with compelling evidence that early ART uptake by co-infected patients reduces mortality [38], there remain widespread controversies on the potential of this intervention to achieve high ART uptake among co-infected patients in SSA.

The summary estimate for ART uptake was suboptimal, but this finding should be interpreted with caution mainly because of the high between-study variability. ART initiation initially depended on CD4 cell count even in TB/HIV co-infected patients but since 2015, WHO has recommended ART initiation irrespective of CD4 count [39]. If we had used year of ART initiation policy change to categorise the studies into groups (before and after policy change) and then do subgroup analysis by year, it would not have been sensible to conclude that any observed subgroup effects are attributable to ART initiation policy change. This is because we observed an extreme paucity of data from subjects under the new (‘test and treat’) ART initiation policy. Even though a good proportion of the studies were published after the WHO had issued new guidelines on ART initiation and TB/HIV collaborative activities, most studies were still based on the old policies. This may suggest that there have been serious delays in implementing the new policies which, relative to the old policies, are expected to reduce delays in ART initiation. And indeed, in 2019, data from WHO revealed that only 11% of low-and middle-income countries were implementing the ‘test and treat’ policy [40]. These notwithstanding, we observed a tendency for studies published after the revised TB/HIV collaborative guidelines to favour much higher ART uptake than studies published before the revised guidelines and summary estimates from subgroup analysis based on TB/HIV collaborative activities guidelines revealed a much higher pooled ART uptake in the era of the revised guidelines. These may indicate that despite the suboptimal pooled ART uptake of 53% and apparent delays in implementing new policies, there has been an overall net improvement in ART uptake since 2012.

It is also worth noting that most of the studies in this review were retrospective and subgroup analysis showed that pooled ART uptake was highest for retrospective studies. However, these studies tended to rely on routine programmatic data, and the quality of such data in low-income settings is often questionable. Prospective cohort studies are expected to provide more reliable data and subgroup analysis showed that pooled ART uptake for these studies was much lower than that for retrospective studies, but the same as the overall pooled ART uptake of 53%. This pooled uptake is much lower than the ART uptake in the general HIV-infected population in SSA in 2019 (76% for women and 62% for men) [41]. A previous meta-analysis reported an ART uptake rate of 83%. However, the external validity of the review was limited by the thin data (just 9 studies were eligible for the review), and two-thirds of the studies were from South Africa [42]. In view of these, studies employing robust methods on a largescale to assess the effectiveness of integrated treatment programmes in increasing treatment outcomes among co-infected patients in SSA are clearly needed.

The most frequently described socioeconomic and patient-level barriers to ART uptake were stigma, low socioeconomic status, and youth. Perceived and anticipated stigma associated with HIV and TB may cause patients not to disclose their HIV status or access ART and in some communities, specific subgroups such as young males and people of low socioeconomic status are more subject to stigma [31]. Previous reports have highlighted the huge contribution of HIV and TB-related stigma to poor health seeking behaviours including HIV status denial and poor uptake of ART [43–45] as well as negative coping strategies such as alternative medicine [31, 45] as captured in this review. A previous systematic review also reported these factors as important barriers to the uptake of ART in the prevention of maternal-to-child transmission of HIV in sub-Saharan Africa [46]. Psychosocial support of various types have been previously highlighted as primordial to reduce stigma, improve health seeking behaviours and disclosure patterns, and empower patients socioeconomically [45] so as to increase uptake of ART. A previous systematic review in the general HIV population proposed increased in confidentiality in low-and middle-income settings where stigma persists [47]. Promoting strategies to engage HIV-infected persons and their families in the treatment and care of HIV, for example, through community-based activities like psychosocial support groups and community-ART delivery groups (CARGs) run by HIV-infected persons [48] can be effective in reducing stigma, and improving uptake of and adherence to treatment. Household counselling is also an important strategy to engage both patients and their families in reducing stigma. It is worth noting that decentralized treatment delivery strategies such as CARG are effective in reducing stigma, transport cost, hospital waiting time and congestion and increasing accessibility/nearness to treatment, which are important factors that determine access to HIV treatment in low-income settings [49–52]. A study by Chileshe et al. included in this review demonstrates how the persons living with HIV support group successfully linked a patient to treatment [28]. A study by Njozing et al. also included in this review reveals the huge role of these groups in counselling, home visits and strengthening staff capacity [35]. The importance of these community-driven approaches in improving uptake of ART in the contexts of treatment of HIV in the general population [53] and the prevention of mother-to-child transmission of HIV [46] have also been reported by previous systematic reviews of studies in sub-Saharan Africa [46]. Economic barriers remain a major challenge to initiate treatment and strategies to alleviate the economic burden of treatment have been also highlighted in a systematic review in low-and middle-income settings [47].

Shortages in HIV commodities as well as limited staff capacity and infrastructure tend to emerge as important system-level therapeutic challenges of TB/HIV treatment in low-income settings [54, 55] and can seriously delay uptake of ART and lead to attrition from ART [54]. This concurs with findings from a previous systematic review which revealed that health system issues such as staffing impair uptake of ART in the prevention of mother-to-child transmission of HIV in sub-Saharan Africa [46]. In a recent large qualitative study in Ghana, infrastructural challenges and lack of staff capacity (notably, understaffing) were highlighted as important challenges in providing treatment commodities in integrated treatment of TB and HIV [56]. These suggest that providing training to adequate number of designated staff, ensuring they have the necessary accompanying treatment resources (through a robust procurement and supply chain) including therapeutic guidelines, developing short and concise enrolment procedures and adequate treatment infrastructure could strengthen the health system, improve service quality, improve outcomes (including those related to drug-related challenges) and consequently contribute in promoting uptake of ART. Designated staff should receive sufficient mentorship and motivation through which they could be encouraged to devote extra time during weekends and emergency circumstances, collaborate in a multidisciplinary team and offer quality services for optimal treatment outcomes. The provision of mentorship to staff engaged in integrated TB/HIV treatment has been previously encouraged as a strategy to improve uptake of ART in co-infected patients in low-income settings and could therefore be included in TB/HIV integrated treatment protocols in order to strengthen staff capacity and enhance initiation of ART [57]. In a previous systematic review, Ahmed and colleagues also emphasized on fear of side effects as a barrier to ART uptake in low-and middle-income settings and highlighted the pivotal role of continually providing information on the health benefits of ART and low side effects of current regimens [47].

Major disruptions in TB/HIV services caused by the COVID-19 pandemic have further demonstrated merits in providing integrated and decentralized treatment services to persons infected with TB and HIV [58]. While some innovations to maintain service delivery have been reported [58], it is worth noting that inadequate knowledge of pre-existing barriers to treatment compliance within integrated TB/HIV programmes prior to the pandemic may have hugely impaired the formulation of adequate strategies of adaptation to limit low ART uptake and attrition from treatment during the pandemic. This is especially true of African settings where TB and HIV services already faced serious challenges before the pandemic occurred.

This study is not void of limitations. We excluded grey literature and studies published in languages other than English and this possibly reduced the variety of barriers and enablers that were captured. Furthermore, the high variability between studies with estimates of uptake negatively affect the validity of the summary estimates of ART uptake, so these results should be interpreted with caution. Additionally, the data supporting each theme on barriers to and enablers of ART uptake were generally thin. Also, whilst this review provides evidence to improve the quality of integrated TB/HIV treatment services, it is limited to ART uptake in patients concurrently or later diagnosed with HIV and does not report TB-related outcomes.

Nonetheless, this study contributes to addressing the crucial lack of data regarding opportunities for improving ART uptake in the domain of integrated treatment for HIV and TB in SSA, the region with the highest burden of HIV/TB co-infection worldwide. Moreover, previous reports in the region generally provide quantitative data on coverage and functionality of integrated treatment, with little attention towards qualitative data which include major drivers of suboptimal treatment outcomes. This study employed a systematic and robust approach to fill the knowledge gap in facilitators of uptake of ART in integrated HIV/TB treatment services. Consequently, the evidence generated is expected to be of sufficiently high quality to adequately inform the formulation and implementation of integrated HIV/TB treatment policies in SSA. Based on our findings, ART uptake in programmes integrating TB/HIV treatment services in SSA is suboptimal and this suggests the important need to review strategies to improve ART uptake within these programmes. By exploring and synthesizing evidence on a broad range of factors that determine uptake of ART in integrated HIV/TB treatment, this review has highlighted key intervention points to improve ART uptake and improve treatment outcome in programmes integrating treatment of HIV and TB in SSA.

## Conclusion

The pooled ART uptake in this study was suboptimal even when compared to ART uptake in general HIV population. This suggests that programmes integrating treatment of TB and HIV in SSA do not, in general, achieve high uptake of ART among co-infected patients. Nonetheless, recent studies tended to show much higher uptake than older studies and this may indicate an overall improvement in ART uptake in recent times. The potential to increase ART uptake among co-infected patients in SSA through these programmes will remain elusive unless a plethora of barriers are addressed using effective interventions. The variety of barriers and enablers observed indicates the broad range of perspectives that ought to be considered to improve ART uptake in integrated TB/HIV treatment services in SSA. Nonetheless, the recurrence of some specific system-level (staff capacity and medical supplies), and patient-level (stigma, income, and psychosocial support) determinants reveals key intervention points to improve ART uptake through integrated treatment programmes in SSA.

## Supplementary Information

**Additional file 1.** Search strategy for the systematic review (designed for Medline and re-adapted when searching each database).

**Additional file 2.** PRISMA Checklist.

**Additional file 3.** Subgroup analysis by setting (rural versus urban versus rural and urban).

**Additional file 4.** Subgroup analysis by region of Africa.

**Additional file 5.** Subgroup analysis by WHO TB/HIV management guideline.

**Additional file 6.** Subgroup analysis by study design.

**Additional file 7.** Subgroup analysis by sample size.

## Data Availability

Not applicable.

## Declarations

### Patient and public involvement

There was no patient or public involvement in the conduct of this study.
