# Supplementary material for: Barriers to and enablers of uptake of antiretroviral therapy in integrated HIV and tuberculosis treatment programmes in sub-Saharan Africa: a systematic review and meta-analysis
Source: AIDS Res Ther. 2021 Nov 16;18:85. doi: 10.1186/s12981-021-00395-3 (PMC8594459; doi:10.1186/s12981-021-00395-3)
Supplement: Supplementary file 1 — Additional file 1. Search strategy for the systematic review (designed for Medline and re-adapted when searching each database). [file 12981_2021_395_MOESM1_ESM.docx]

**Search strategy for the systematic review**

| Search # | Search words |
| --- | --- |
| 1 | (Antiretroviral therapy OR ART) AND (Uptake OR start* OR initiat*) |
| 2 | (Integrat* OR joint OR collaborat* OR concurrent) AND (Tuberculosis OR TB) AND (HIV OR AIDS) AND (treat* OR therap* OR care OR service) |
| 3 | Barrier OR challenge OR drawback OR limitation |
| 4 | Enabl* OR facilitat* OR opportunit* OR driver |
| 5 | Africa OR Algeria OR Angola OR Benin OR Botswana OR Burkina Faso OR Burundi OR Cameroon OR Cape Verde OR Central African Republic OR Chad OR Comoros OR Congo OR Democratic Republic of Congo OR Djibouti OR Egypt OR Equatorial Guinea OR Eritrea OR Ethiopia OR Gabon OR Gambia OR Ghana OR Guinea OR Guinea Bissau OR Ivory Coast OR Cote d’Ivoire OR Jamahiriya OR Jamahiryia OR Kenya OR Lesotho OR Liberia OR Libya OR Libia OR Madagascar OR Malawi OR Mali OR Mauritania OR Mauritius OR Mayotte OR Morocco OR Mozambique OR Mocambique OR Namibia OR Niger OR Nigeria OR Principe OR Reunion OR Rwanda OR Sao Tome OR Senegal OR Seychelles OR Sierra Leone OR Somalia OR South Africa OR St Helena OR Sudan OR Swaziland OR Tanzania OR Togo OR Tunisia OR Uganda OR Western Sahara OR Zaire OR Zambia OR Zimbabwe OR Central Africa OR Central African OR West Africa OR West African OR Western Africa OR Western African OR East Africa OR East African OR Eastern Africa OR Eastern African OR North Africa OR North African OR Northern Africa OR Northern African OR South African OR Southern Africa OR Southern African OR subSaharan Africa OR sub-Saharan African OR sub-Saharan Africa OR sub-Saharan African |
| 6 | #1 AND #2 AND #3 AND #5 |
| 7 | #1 AND #2 AND #4 AND #5 |
