# Supplementary material for: Barriers to and enablers of uptake of antiretroviral therapy in integrated HIV and tuberculosis treatment programmes in sub-Saharan Africa: a systematic review and meta-analysis
Source: AIDS Res Ther. 2021 Nov 16;18:85. doi: 10.1186/s12981-021-00395-3 (PMC8594459; doi:10.1186/s12981-021-00395-3)
Supplement: Supplementary file 6 — Additional file 6. Subgroup analysis by study design. [file 12981_2021_395_MOESM6_ESM.docx]

**ART uptake**

**Supplementary file 6: Forest plot showing pooled ART uptake stratified by study design.** *The dashed line on the Forest plot represents the overall pooled estimate. The grey squares and horizontal lines represent the individual study ART uptakes and their 95% confidence intervals. The size of the grey square represents the weight contributed by each study in the meta-analysis. The diamond represents the pooled estimate and its 95% confidence intervals.*
